# Editorial: The emotional antecedents and consequences of social rejection

**DOI:** 10.3389/fpsyg.2023.1279894

**Published:** 2023-09-21

**Authors:** Richard S. Pond, John A. Terrizzi, Shanmukh V. Kamble

**Affiliations:** ^1^Department of Psychology, University of North Carolina Wilmington, Wilmington, NC, United States; ^2^Department of Psychology, Texas Woman's University, Denton, TX, United States; ^3^Department of Psychology, Karnatak University, Dharwad, India

**Keywords:** social rejection, social exclusion, social pain, ostracism, stigma, emotion

## Introduction

Social scientists have long argued that the need to belong is a central feature of human psychology and a cross-cultural human universal (Baumeister and Leary, 1995). Not only does social connection provide us with numerous survival benefits (e.g., aid in building shelter, providing defense, etc.), but we also tend to suffer from a host of harmful psychological and physiological consequences when our need to belong is thwarted (see DeWall and Bushman, 2011, for a review). As a result, social rejection is an aversive experience that can be strategically employed to inflict harm and punishment.

**Figure 1:**
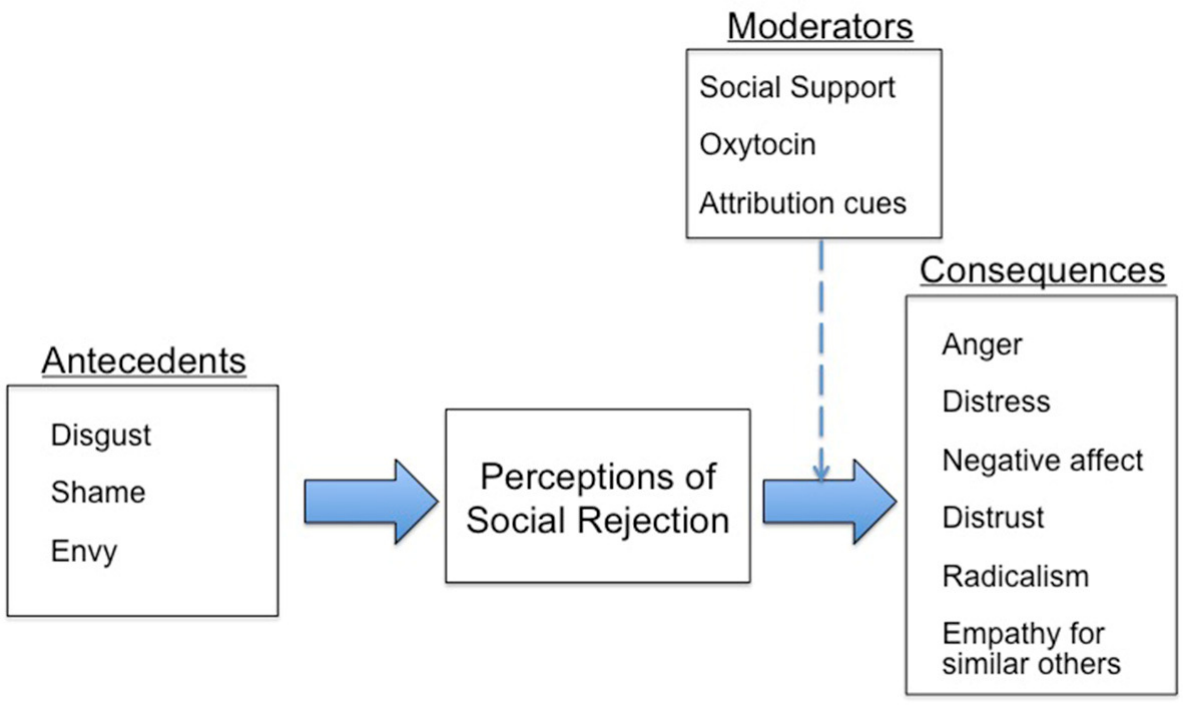
Examples of emotional antecedents, consequences, and moderators identified in the present collection of manuscripts.

Social rejection experiences have both emotional antecedents and consequences. That is, strong emotional experiences (e.g., anger, disgust, etc.) within actors may provoke them to engage in social rejection behaviors, whereas targets of rejection may suffer various emotional consequences (e.g., anger, sadness, emotional numbness, etc.). Thus, the role that emotion plays in social rejection is not simple; it is multifaceted (see Figure 1). Negative emotions can provoke social rejection and, reciprocally, the rejection experience can evoke negative emotions in those who are rejected. Positive emotions, however, may act as a buffer or shield that insulates us from the deleterious consequences of rejection.

The goal of the present Research Topic was to provide an opportunity for contributors to present a current overview of the recent theoretical and methodological advances in the areas of belongingness, social rejection, stigma, and emotion in order to shed light on the multifaceted relationship between emotional experience and social rejection. In addition, we hoped to generate discussion about implications for future work in the area, as well as practical applications, and to identify critical gaps left to be explored.

As a result of the work of 22 authors and 21 reviewers, nine manuscripts were published in this Research Topic of *Frontiers in Psychology* (*The emotional antecedents and consequences of social rejection*; participating sections included: *Personality and social psychology, emotion science*, and *evolutionary psychology*) between May 11, 2022 and June 16, 2023. These manuscripts varied in form and methodological approach, including psychophysiological assessments (Park et al.; Yin and Lee), experience-sampling techniques (Wang and Li), self-report methods (Park and Joshanloo; Pfundmair and Mahr), behavioral experiments (Dvir and Nagar; Knausenberger et al.; Yaakobi) and a mini-review (Terrizzi et al.). The basic themes of the contributions fall into three categories: 1. Emotional antecedents to social rejection, 2. Emotional consequences of social rejection, and 3. Emotional buffers or resiliency factors to the consequences of rejection. These themes are discussed further below.

### Emotional antecedents

Two papers within the Research Topic focused more directly on the antecedents of rejection. First, Terrizzi et al. summarized evidence that suggests that disgust and shame (i.e., a self-directed form of disgust) are both key antecedents to the rejection experience, due to their roles in promoting stigmatization among actors and self-isolation among targets, respectively. In addition, Wang and Li used experience-sampling techniques to identify envy toward a target as another key antecedent to social rejection.

### Emotional consequences

A number of papers within the Research Topic emphasized the emotional consequences of rejection, using a variety of different methods for inducing rejection. For instance, Dvir and Nagar explored sexual objectification as a partial form of ostracism (i.e., when one's body is the focus of another's attention as opposed to one's internal thoughts and feelings). They showed that sexual objectification tended to reduce the incidence of victim-blaming among women, because it increased empathy for other victims of objectification. These effects, however, appeared to be attenuated for women who experienced a form of ostracism that was unrelated to sexual objectification. Knausenberger et al. demonstrated that *phubbing* (i.e., a momentary act of ostracism that occurs when actors divert their attention to their phone instead of their conversation partner) yields negative consequences for mood and trust, similar to more explicit forms of ostracism (e.g., rejection by a group). Likewise, using methods from electroencephalography (EEG), Yin and Lee showed that loneliness primes negatively impacted mood and increased hypervigilance to threat. Further, changes in event-related potentials (ERPs) due to the loneliness primes were negatively associated with prosociality. Finally, Pfundmair and Mahr revealed that feelings of social exclusion as a function of COVID-19 containment policies were associated with increased radicalism, partly as a means of re-establishing feelings of control.

### Emotional buffers

Three papers within the Research Topic addressed factors that may buffer or reduce the emotional distress of social rejection. Park and Joshanloo observed, among a South Korean sample, that perceived social support shielded participants from the negative impact that ethnic discrimination (a form of social rejection) has on mood and wellbeing. Park et al. explored reactions to out-group acceptance and rejection among ethnic/racial minoritized participants. They found that the administration of intranasal oxytocin amplified favorable responses (in terms of cardiovascular reactivity, cooperative behavior, and partner perceptions) among Black participants who received positive feedback from White partners. However, intranasal oxytocin also tended to amplify angry reactions to negative feedback from White partners. Finally, Yaakobi explored the mitigating effects of attributional cues during ostracism recovery. Specifically, participants who were able to make unstable and external attributions (e.g., being left out because of “bad luck”) showed less distress post-ostracism compared to participants who made stable and internal attributions (e.g., “it's because of my personality”).

## Concluding remarks

Our hope in presenting this special topic for *Frontiers in Psychology* was to shed light on the multifaceted nature of the relationship between emotional experience and social rejection. The present collection of papers demonstrated the fundamental connection between our emotional and social lives. Further, they each highlighted new directions for future research, particularly in regards to resiliency factors that may protect against belongingness threats. We would like to thank all the authors and reviewers who contributed to the success of this project.

## Author contributions

RP: Writing—original draft. JT: Writing—review and editing. SK: Writing—review and editing.

## References

[B1] Baumeister R. F. Leary M. R. (1995). The need to belong: desire for interpersonal attachments as a fundamental human motivation. Psychol. Bull. 117, 497–529. 7777651

[B2] DeWall C. N. Bushman B. J. (2011). Social acceptance and rejection: the sweet and the bitter. Current Direct. Psychol. Sci. 20, 256–260. 10.1177/0963721411417545

